# Hyperparameter Optimization for Atomic Cluster Expansion Potentials

**DOI:** 10.1021/acs.jctc.4c01012

**Published:** 2024-11-06

**Authors:** Daniel F. Thomas du Toit, Yuxing Zhou, Volker L. Deringer

**Affiliations:** Inorganic Chemistry Laboratory, Department of Chemistry, University of Oxford, Oxford OX1 3QR, U.K.

## Abstract

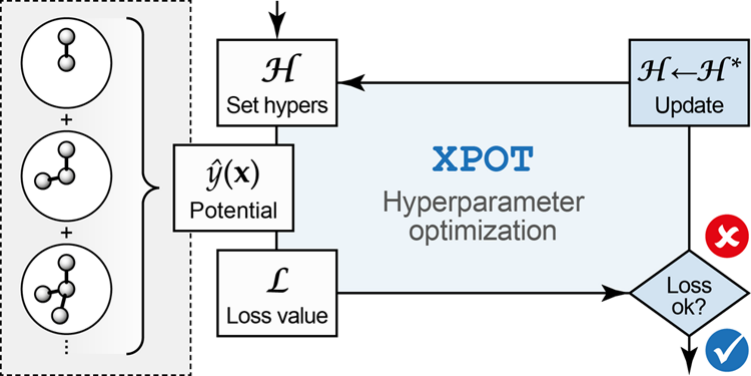

Machine learning-based interatomic potentials enable accurate materials simulations on extended time- and length scales. ML potentials based on the atomic cluster expansion (ACE) framework have recently shown promising performance for this purpose. Here, we describe a largely automated computational approach to optimizing hyperparameters for ACE potential models. We extend our openly available Python package, XPOT, to include an interface for ACE fitting, and discuss the optimization of the functional form and complexity of these models based on systematic sweeps across relevant hyperparameters. We showcase the usefulness of the approach for two example systems: the covalent network of silicon and the phase-change material Sb_2_Te_3_. More generally, our work emphasizes the importance of hyperparameter selection in the development of advanced ML potential models.

## Introduction

Machine learning (ML) approaches to interatomic potential fitting are now widely used in computational chemistry.^1−5^ ML potentials provide surrogate models for quantum-mechanical (QM) potential-energy surfaces at a fraction of the cost, providing longer-time scale and larger-length scale simulations than would be accessible with direct QM methods, while maintaining comparable accuracy. As a result, they are now becoming widespread tools to simulate materials and molecules, with applications ranging from the structure of disordered solids^6,7^ to modeling nuanced effects such as anharmonic phonons^8^ or noncollinear magnetism in iron,^9^ as well as materials not yet synthesized.^10^

Many methods for fitting ML potentials have now been developed, from the early Behler–Parrinello neural-network^11,12^ and Gaussian Approximation Potential (GAP)^13−15^ models to more recent atomic cluster expansion (ACE) potentials,^16−18^ as well as graph-neural-network architectures.^19−22^ Each of these methods has different characteristics: for example, graph-based architectures currently define the state-of-the-art in terms of accuracy, but inference (prediction) is still relatively expensive for large systems.^23,24^ Our focus herein is on ML potentials that are based on ACE descriptors and fitted using the PACEmaker software by Drautz and co-workers,^17,18^ which have shown to provide efficient and accurate predictions, e.g., for data from ref (25).^17^

Despite their popularity, ML potentials pose challenges because they largely lack physically motivated functional forms. First, they require high-quality reference data:^26^ recent hand-crafted ML potentials for carbon^6^ and Ge–Sb–Te^7^ each used more than 300,000 atomic environments for training; for potentials encompassing many different elements, the data set sizes can easily range in the millions.^27,28^ Second, ML potentials require well-chosen hyperparameters—those parameters which must be chosen before fitting starts, and thus determine the nature of the fitting. For mathematically complex ML models in particular, the effects of changing hyperparameters are not always immediately clear. For example, in the ACE approach we use here, the prefactors and exponents of atomic property terms which contribute to the energy (see Methods section below) can be difficult to tune manually. It would therefore be desirable to explore the space of hyperparameters automatically and to optimize them with minimal user input.

We previously showed how hyperparameter optimization for spectral neighbor analysis potential (SNAP; ref (29).) models can improve their prediction accuracy without increased cost at runtime.^30^ Conversely, with poorly chosen hyperparameters, accuracy and robustness may suffer. We note that more widely, key advances have been made in recent years in terms of choosing hyperparameters for ML potentials.^30−34^

**Figure 1:**
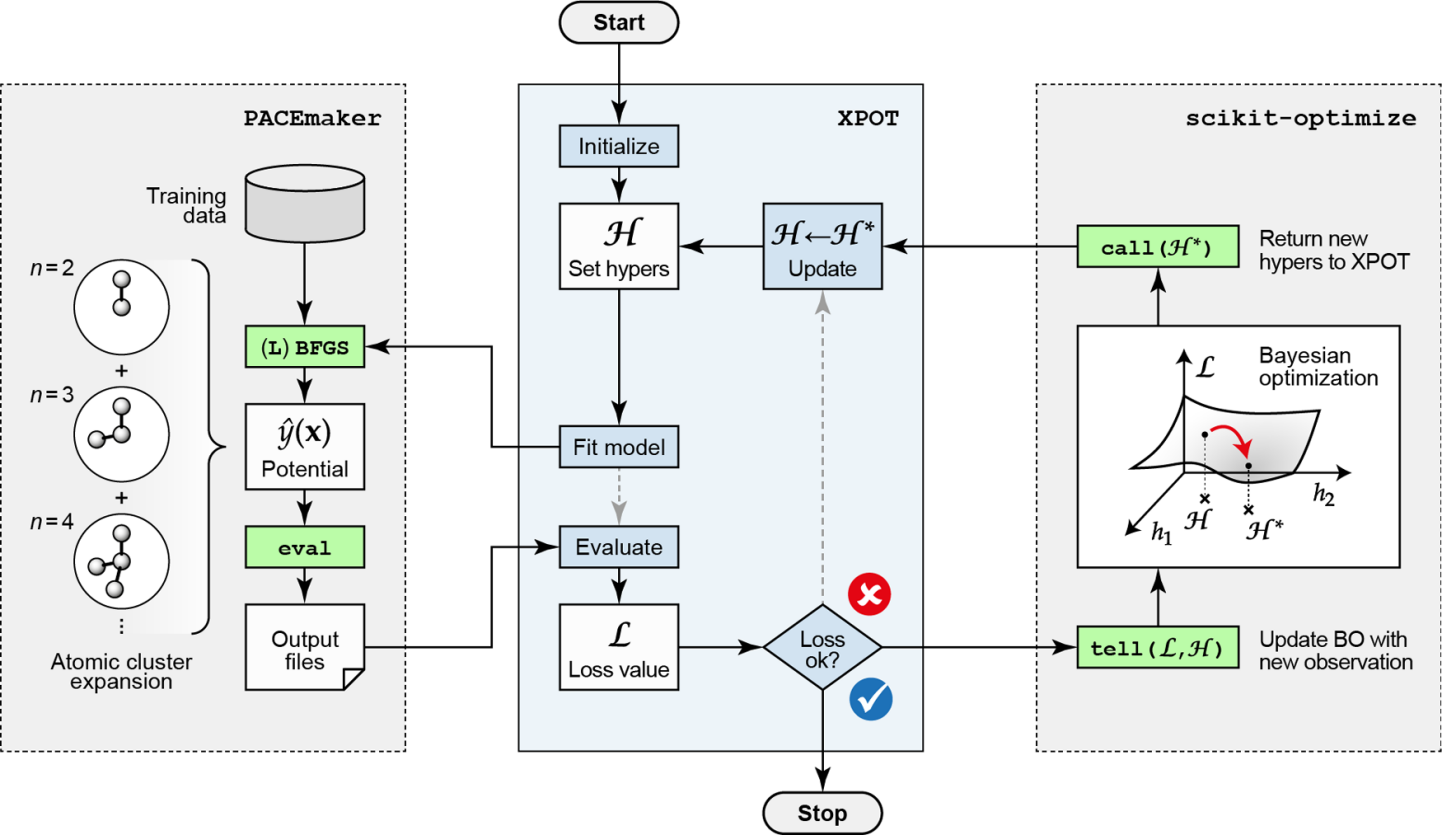
Overview of the methodology for automated optimization of ACE potentials, showing a simplified flowchart of the computational tasks involved. The core functionality of XPOT is highlighted in the central box in blue, and external calls to PACEmaker (left) and scikit-optimize (right) are indicated.

In this work, we study the effect of hyperparameter optimization for ACE ML potentials, fitted to existing high-quality data sets for silicon^35^ and Sb_2_Te_3_.^7^ We investigate both numerical and structural predictions and benchmark the performance of the resulting ACE models. For this purpose, we extended our automated hyperparameter optimization package, “Cross-platform optimizer for machine learning interatomic potentials” (XPOT),^30^ to include support for ACE potentials fitted with PACEmaker(18) (Figure 1). In doing so, we emphasize that XPOT enables efficient transfer between fitting frameworks, here from GAP to ACE—giving access to potentials which are cheaper to run while maintaining comparable accuracy for the same training data.

## Methods

### Atomic Cluster Expansion

The atomic cluster expansion (ACE) produces a complete set of basis functions which span the space of local atomic environments, as described in refs (16−18). In recent years, the ACE framework has shifted the Pareto front for cost and accuracy of ML potentials^17^ and has underpinned the development of state-of-the-art interatomic potential models.^6,36−38^ For example, a general-purpose ACE potential for carbon^6^ outperforms previously reported models^39^ in terms of both accuracy and speed, and an ACE model for Si–O was shown to accurately describe complex nanoscale structural features in this key binary system.^37^

ACE-based ML models are built from general atomic “properties”, φ_*i*_, expanded across body-ordered functions from the set of neighbors of each atom. Any given property of atom *i* (index *p*) can therefore be expanded as a function of this atom’s local environment:
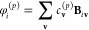
1where the expansion coefficients *c*_**v**_^(*p*)^ and basis functions (**B**_*i***v**_) share multi-indices *v* which describe the list of basis functions in a cluster. The potential energy of the *i*th atom—typically the prediction target for ML potential models—can be described as a combination of several atomic properties. In the simplest case, the atomic energy, ε_*i*_, depends on only a single value:
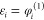
2which leads to a linear model. However, ACE potentials are not limited to using a single property: introducing nonlinear behavior through a Finnis–Sinclair-like embedding, for example, results in
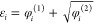
3where the two properties are generalizations of the pairwise repulsion and density of the widely used Finnis–Sinclair potential.^40^ This approach can be further extended to an arbitrary number of *P* atomic properties (*p* = 1, ..., *P*) which all enter as arguments into a nonlinear function, denoted *F*:
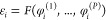
4where *i* again is the atomic index. For example, in ref (37)., it was shown that a complex functional form (*P* = 8) outperformed linear and Finnis–Sinclair type embeddings for ACE models for the Si–O system while only leading to a ≈15% increase in computational requirements. A full systematic study of the number, exponents, etc. of the terms that enter *F* is yet to be reported, and it is likely that there is some scope for optimizing nonlinear ACE models in this regard.

### XPOT

We use our Python package, XPOT,^30^ to fit optimized ACE models. XPOT automates the optimization of hyperparameters within ranges specified by the user, by iteratively minimizing a combined energy and force loss function which is defined as
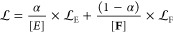
5In this expression, square brackets indicate the units (in the present work, we use energies in eV  and forces in eV Å^–1^ respectively), emphasizing that the loss function, , itself is dimensionless. By adjusting the relative energy and force loss weighting, controlled by the single parameter α, the potential fit can be optimized toward the desired characteristics. XPOT evaluates  over a validation data set, which is not used in training the model.^30^

The energy contribution to the loss function, , is given by
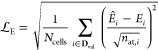
6where *N*_cells_ is the number of structures in the validation set, **D**_val_, and *n*_at,*i*_ is the number of atoms in the *i*th structure from that set. The energy prediction of the ACE model is *Ê*_*i*_, whereas the “correct” reference energy in the validation set is *E*_*i*_. The difference between the two is divided by —not *n*_at,*i*_—in order to weight the contribution of energy errors from each structure equally, regardless of the number of atoms in any given validation-set structure.^41^

The force contribution to the loss function,
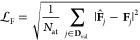
7is determined by measuring the magnitude of the error between the predicted and reference force vectors. The force error is averaged over all *N*_at_ atoms in the data set.

### Implementation of ACE Support in XPOT

Figure 1 lays out the workflow of XPOT, outlining the distribution and ordering of tasks within the hyperparameter optimization loop. This figure specifically relates to PACEmaker and ACE potentials, but the XPOT workflow is similar for other fitting methods. XPOT interfaces both to potential fitting software (left-hand side in Figure 1) and to the Bayesian optimization (BO) interface of scikit-optimize (right-hand side). The latter supports discrete and continuous variable optimization, as discussed in ref (30). The user-defined hyperparameter ranges are parsed, before potentials are fitted iteratively, and new hyperparameters are chosen through BO predictions until the loss reaches the desired value, or the maximum number of iterations is reached. A full description of the XPOT code can be found in ref (30).

To begin the process, the user initializes the optimization in Python, specifying an input script containing the hyperparameters to be optimized. XPOT parses this input and uses scikit-optimize to set values for each iteration. Then, PACEmaker is invoked, fitting the ACE potential model, and calculating the errors on both the training and validation data sets. XPOT parses the output files and converts the prediction errors into a single loss function,  (see eq 5). At this stage, if optimization is determined to be finished, XPOT exits, otherwise calling another estimation of the loss surface and updating the hyperparameters to continue the process.

While the optimization process in XPOT is agnostic to the specific fitting methodology, file parsing between XPOT and the desired fitting software must be implemented. Here, to reduce compute-time and installation requirements, while evaluation of the loss function is done by XPOT, we use the detailed per-structure predictions on the validation data set (performed by PACEmaker after fitting) as the errors to calculate the loss. As a result, unlike XPOT’s interface to fitsnap, LAMMPS is not required for optimization of ACE potentials. GPU fitting with PACEmaker is supported in XPOT, and can be controlled by specifying the desired CUDA device at runtime using environment variables.

### Data Sets

We briefly describe the data sets that we use in the present study for fitting and validating potentials. For details, we refer to the cited original publications; for an overview of validation techniques for ML potentials more generally, we refer to ref (41).

In the case of silicon, we use three different data sets for specific purposes. First, we take the training data set for the Si-GAP-18 general-purpose potential by Bartók et al.^35^ as an example of a highly developed and largely handcrafted data set. We fit ACE models to this data set using XPOT, and we use the corresponding test set for validation (both taken from ref (35).). Second, we use DFT-labeled snapshots from a GAP-MD simulation described in ref (42). (referred to as “MQ-MD” in the following). This data set contains diamond-type supercells with vacancy defects, liquid, and amorphous structures, as well as transitions between these phases. The structures were generated with Si-GAP-18-driven MD and subsequently labeled with single-point DFT computations.^42^ Finally, we test our XPOT-fitted models on random structure search (RSS) configurations from ref (43). These allow us to test the potentials’ ability to describe structures different from those on which they were trained.

**Figure 2:**
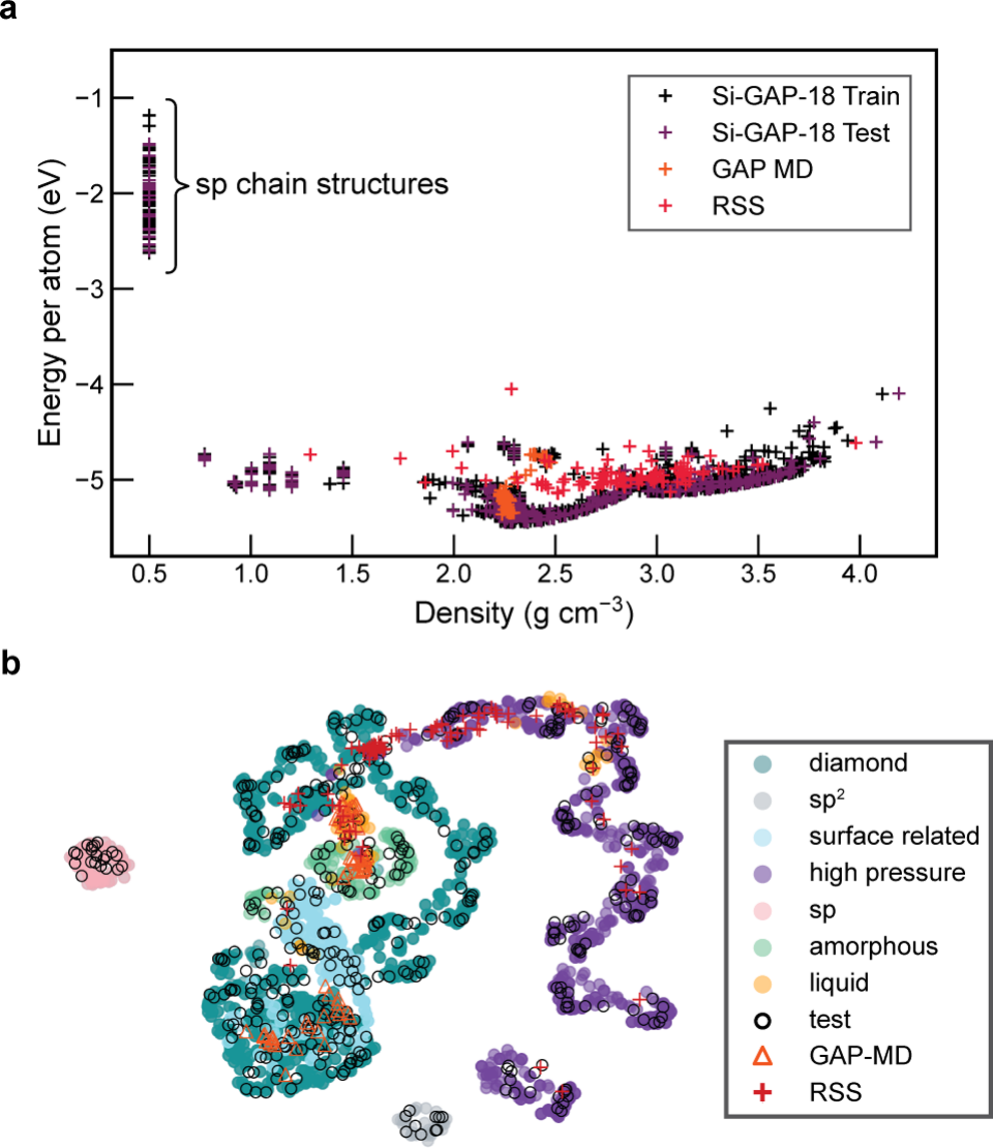
Visualization of the data for Si used in this work. (a) An energy–density plot showing the heterogeneous nature of the Si-GAP-18 training data set (ref (35).), with the “sp” chain structures highlighted. (b) UMAP embedding of the averaged ACE vectors for structures in the silicon data sets, highlighting the overlap in local atomic environments between structures. The categories are partly simplified compared to ref (35).: here, for example, “diamond” also includes defective diamond-type Si structures.

In Figure 2a, we characterize the various training and testing sets used by plotting the energy against the mass density for each structure. The bulk structures mainly have densities relevant to amorphous, crystalline, and liquid silicon, with sp-like chain structures constituting the data set entries at very low density and high energy. Despite the difference in the composition of the RSS, MQ-MD, and Si-GAP-18 test data sets, the structures seem broadly related when compared in this plot.

In Figure 2b, we show a similarity map, obtained by UMAP dimensionality reduction^44^ on the ACE vectors of the atomic environments averaged for each structure. This map indicates distinct regions, mirroring the varied structure types included in the data set. By color-coding the points in the map according to the configuration types defined in the Si-GAP-18 training data set,^35^ we can visualize the isolated nature of the low-coordinate (“sp” and “sp^2^”) and high-pressure structures (β-Sn-type and simple hexagonal), and the relation between the various phases included. Additionally, we can visualize the coverage of the various test sets and their similarity to the training data. The RSS data set includes entries that (although clearly higher in energy; cf. Figure 2a) resemble diamond-like, high-pressure, and liquid-like structures. Finally, we note that all surface-related structures (slab models) in the Si-GAP-18 training set are based on the diamond-type form, and so the similarity of these structures to bulk diamond-type Si is expected.

The data set used in our study of Sb_2_Te_3_ is taken from the GST-GAP-22 data set.^7^ In this case, we remove all structures which include Ge, yielding a data set for the binary Sb–Te system. We randomly split this data set into train and test data (80:20), for each configuration type as defined in the data set, such as “crystalline”, “aimd”, and “liquid”. This way, we can ensure that the validation data span a wide range of structure types, and reduce the possibility of optimizing overly toward a single subgroup of the data set.

## Results and Discussion

### Silicon(I): Numerical Performance

**Figure 3:**
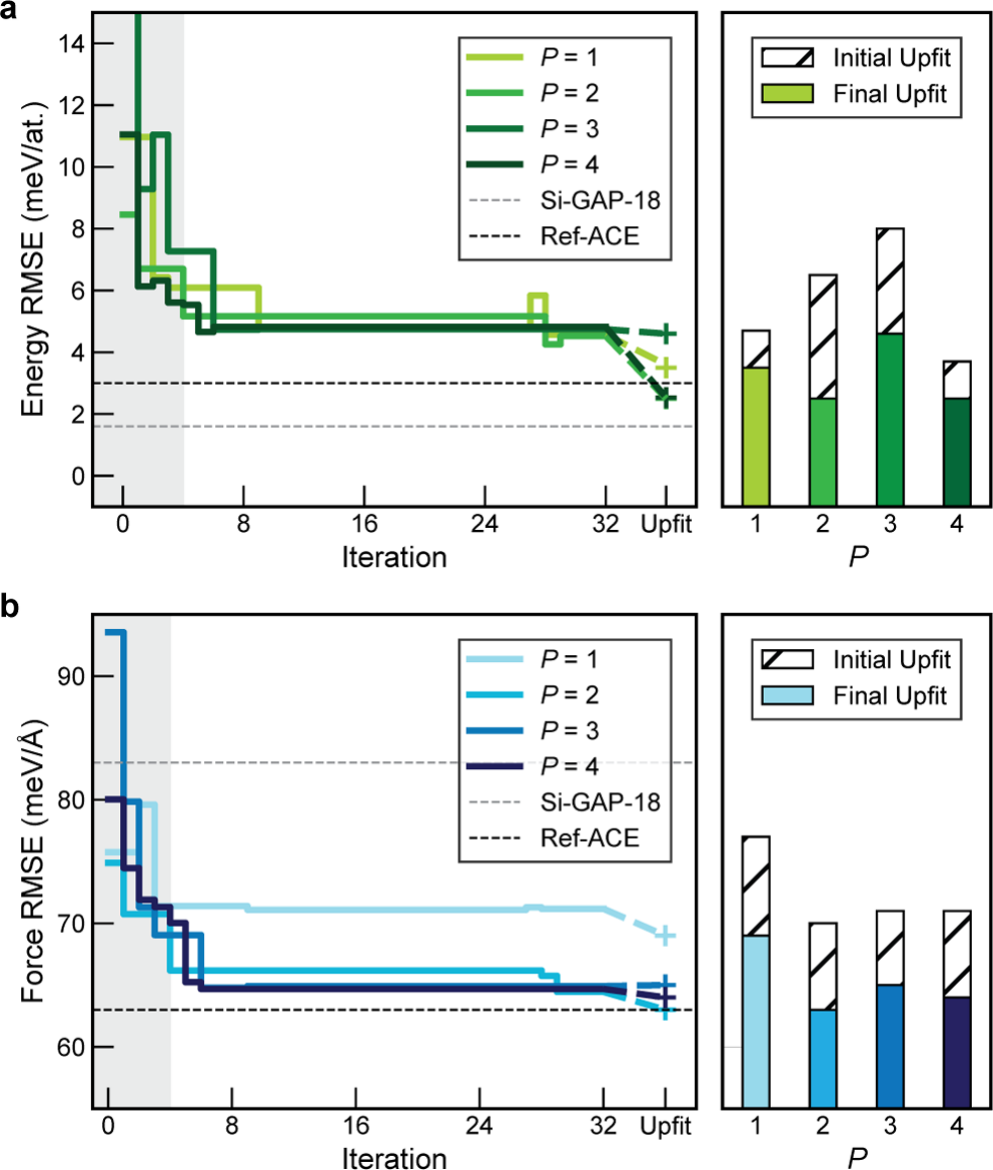
Evolution of energy and force errors for silicon ML potentials through iterative optimization using XPOT. We show (a) the per-atom energy RMSE and (b) the force component RMSE across iterations. Errors are evaluated on the Si-GAP-18 test set from ref (35). On the left, the gray region indicates the initial sampling stage of optimization (see ref (30).). The bar charts show the errors on the test set for “upfitted” potentials, as described in the text: both for the best potentials from the initialization protocol (hatched), and the best potentials after Bayesian optimization (solid). *P* refers to the number of atomic properties, as defined in the Methods section (cf. eq 4). The data for *P* = 3 and *P* = 4 refer to the “-3F” and “-4F” potentials from Table 1, respectively.

We fitted ACE potentials to the general-purpose Si-GAP-18 training data set from ref (35). Previously, Lysogorskiy et al. fitted a linear potential to the same data,^17^ and several nonlinear models (*P* > 1) have been fitted recently for other materials.^6,37^ Here, we create ACE models over the range of 1 ≤ *P* ≤ 4, using fewer basis functions compared to ref (17)., in order to improve efficiency at runtime. Our XPOT-ACE-2 model achieves accuracy comparable to the potential fitted in ref (17). (“Ref-ACE”, Figure 3), taking advantage of optimized hyperparameters and nonlinear ACE models for improved efficiency.

XPOT optimizes hyperparameters by minimizing the combined loss,  (eq 5). As such, XPOT is guided by changes in the errors on the specific validation set (here, the Si-GAP-18 test set^35^). As the MQ-MD and RSS data sets are *not* used as targets in XPOT optimization, we leverage them as distinct benchmarks for accuracy and robustness. The MQ-MD data set from ref (42). is similar in character to the parts of the Si-GAP-18 test set, allowing further accuracy tests on larger structures. In contrast, the RSS data set contains randomly generated structures which are typically higher in energy (Figure 2a). No explicit RSS structures are present in either the Si-GAP-18 test or train sets. Using these two data sets, we can validate the performance of the Si-GAP-18 test set as a general-purpose optimization target.

For each value of *P*, we optimized up to 6 hyperparameters at once, and performed 32 fitting iterations. The first four of these were initialization fits (shaded areas in Figure 3), where a pseudorandom sampling method is used to determine hyperparameters, before Bayesian Optimization (BO) is applied for the remaining iterations. For all models, we optimized φ_*i*_ exponents, cutoff, radial basis function (including radbaseparameter), and dcut (the smoothing distance at the outer limit of the cutoff). We used universal structure weighting—unlike REF-ACE, which includes weightings based on the type of structure in the training set.^17^ This was done to show that even without a “bespoke” approach to weighting data set entries, hyperparameter optimization could improve ML potentials. In future work, we hope to study the effect of weighting techniques to further improve fitting for smaller data sets.

After the optimization, the best potential for each optimization sweep was “upfitted”. By this term, we mean the approach of continuing the fitting process from an existing potential. PACEmaker uses a ratio value, κ, to determine the relative weighting of energy and force errors in the fitting process. A higher value of κ to prioritizes forces when fitting, and a lower κ value prioritizes energies. Upfitting potentials (by varying κ) can potentially improve the accuracy of ACE models.^45^ This process is analogous to the stage two (--swa) setting used in MACE fitting, whereby energy weighting is increased for the final 20% of the fitting process to improve energy errors.^46^ Herein, we first fitted using κ = 0.8, before upfitting the same potential with κ = 0.02. This process was the same for all XPOT-ACE potentials in the present work. After the final potentials were upfitted, we performed numerical validation on two external data sets (see Methods section).

Initially, we fitted potentials with 3000 functions each for all values of *P*, but found an increased likelihood of overfitting for *P* ≥ 3. Table 1 includes results from optimizing potentials for *P* = 3 and *P* = 4 with 3000 functions, highlighting the reduced accuracy and robustness compared to the *P* ≤ 2 potentials. Specifically, the XPOT-ACE-3 potential has become much worse during the upfitting procedure, producing highly unreasonable energy and force errors on the RSS test set. When analyzing the errors per structure for the upfitted XPOT-ACE-3 potential further, it was found that the increased error on the Si-GAP-18 test set comes from two individual structures. We emphasize that these issues would not have become apparent from testing only on the MQ-MD data set, and instead a more wide-ranging analysis including RSS test data reveals that the potential is not sufficiently robust. The nonupfitted potential result from XPOT has a testing error of 7.9 meV at.^–1^—note that while this seems to be an acceptable result, it is already 4× the training error, and thus this potential as well appears to be overfitted.

**Table 1:** Energy and Force RMSE Values of Silicon Potentials, Evaluated on Three Different Test Sets^a^

			energy RMSE (meV at.^–1^)			force RMSE (meV Å^–1^)			
	*P*	# func.	Si-GAP-18-test^35^	MQ-MD^42^	RSS^43^	Si-GAP-18-test	MQ-MD	RSS	MD speed
XPOT-ACE-1	1	3000	3.5	5.1	27.1	69	105	158	47
XPOT-ACE-2	2	3000	2.5	5.0	23.1	63	97	150	46
XPOT-ACE-3	3	3000	318	5.2	>10^6^	300	98	>10^8^	45
XPOT-ACE-4	4	3000	4.8	5.5	62.6	63	99	274	43
XPOT-ACE-3F	3	2000	4.6	4.1	20.5	65	97	139	65
XPOT-ACE-4F	4	1625	2.5	5.4	72.5	64	100	187	80
XPOT-ACE-6827	1	6827	3.0	4.4	34.5	63	104	179	16
REF-ACE^17^	1	6827	3.2	4.3	42.1	77	124	175	16
Si-GAP-18^35^	N/A	N/A	1.6	8.5	34.9	83	139	177	1

a For the XPOT-ACE models, the number of atomic properties, *P*, as defined in eq 4, ranges from 1 to 4. An “F” denotes a potential where the number of functions was optimized by XPOT. XPOT-ACE-6827 is an optimized model using the same number of radial basis functions as the linear ACE potential fitted by Lysogorskiy et al.^17^ (denoted as “REF-ACE” here). The MD speed is given relative to that of Si-GAP-18.

As such, we used XPOT to optimize the number of functions for the *P* ≥ 3 potentials as an additional variable hyperparameter, from 500 up to an upper limit of 2000. By reducing the maximum number of functions, we restricted the flexibility of these models, to optimize toward more robust behavior. We note that XPOT-ACE-3F uses the maximum number of functions (2000), while XPOT-ACE-4F is optimized to 1625 functions. These optimized potentials are denoted with an “F” suffix and the results are included in Table 1. In addition to having improved numerical accuracy and efficiency, these potentials were able to complete melt–quench simulations for 4096-atom cells (Table 2), which the XPOT-ACE-3 and XPOT-ACE-4 potentials were not. However, as the optimization still occurs for the Si-GAP-18 test set, a good performance on RSS data is still not guaranteed, as shown by the high RSS error for XPOT-ACE-4F.

**Table 2:** Energy Per-Atom Differences of Quenched a-Si Structures Relative to (Diamond-Type) Crystalline Silicon^a^

quench rate (K s^–1^)	Δ*E* (meV at.^–1^)			
	XPOT-ACE quenched			
	XPOT	REF	GAP	DFT
10^14^	205(6)	209(6)	215(7)	210(7)
10^13^	183(4)	187(4)	192(4)	185(5)
10^12^	158(3)	161(3)	166(4)	156(4)
10^11^	140(4)	142(5)	146(5)	137(5)

	REF-ACE quenched			
	XPOT	REF	GAP	DFT
10^14^	211(4)	208(4)	218(3)	217(6)
10^13^	190(4)	187(3)	197(4)	185(5)
10^12^	160(4)	158(3)	166(4)	155(5)
10^11^	147(4)	145(4)	153(4)	144(5)

a Structures were quenched and relaxed using the ML potentials in the top row, comparing the differences between XPOT-ACE and REF-ACE.^17^ The standard deviation across the sampled structures is reported using parentheses.

The evolution of energy and force errors is visualized in Figure 3, including the upfitting procedure which is undertaken at the end of the optimization process, as described above. For comparison, we also upfitted the best potentials from the initialization phase, to assess whether the BO part of the process brings additional benefit. We see that across all values of *P*, the final optimized potentials are more accurate on the Si-GAP-18 test set used to evaluate .

The upfitting process for the initial potentials does not always improve testing error, despite the training error reducing throughout the fitting process. This suggests that the initial hyperparameters selected are not providing a generalizable description of silicon, indicated by relatively poor predictive accuracy on unseen environments. Additionally, not all upfitted initial potentials were found to be stable in MD simulations up to 1800 K. We further quantify the accuracy of these potentials in the Supporting Information.

For the nonlinear XPOT-ACE-4F model, we observe that the robustness and accuracy on the test set are sufficient, but it is prone to large errors on other data sets—that is, the model appears to be overfitted. This finding again demonstrates that, as we discussed in ref (30)., the makeup of the validation set is paramount in optimizing a potential toward the desired characteristics. Additionally, setting bounds for the flexibility of the potential reduces the likelihood of overfitting, but the composition of the validation set still directly affects the loss function, and thus the minimum in hyperparameter space toward which the potentials are optimized.

Taking into account the results above, XPOT-ACE-2 seems to offer the best combination of accuracy and robustness among the entries of Table 1. We therefore take this potential forward for further testing, and from here onward we refer to it simply as “XPOT-ACE”.

### Silicon(II): Physics-Guided Validation

**Figure 4:**
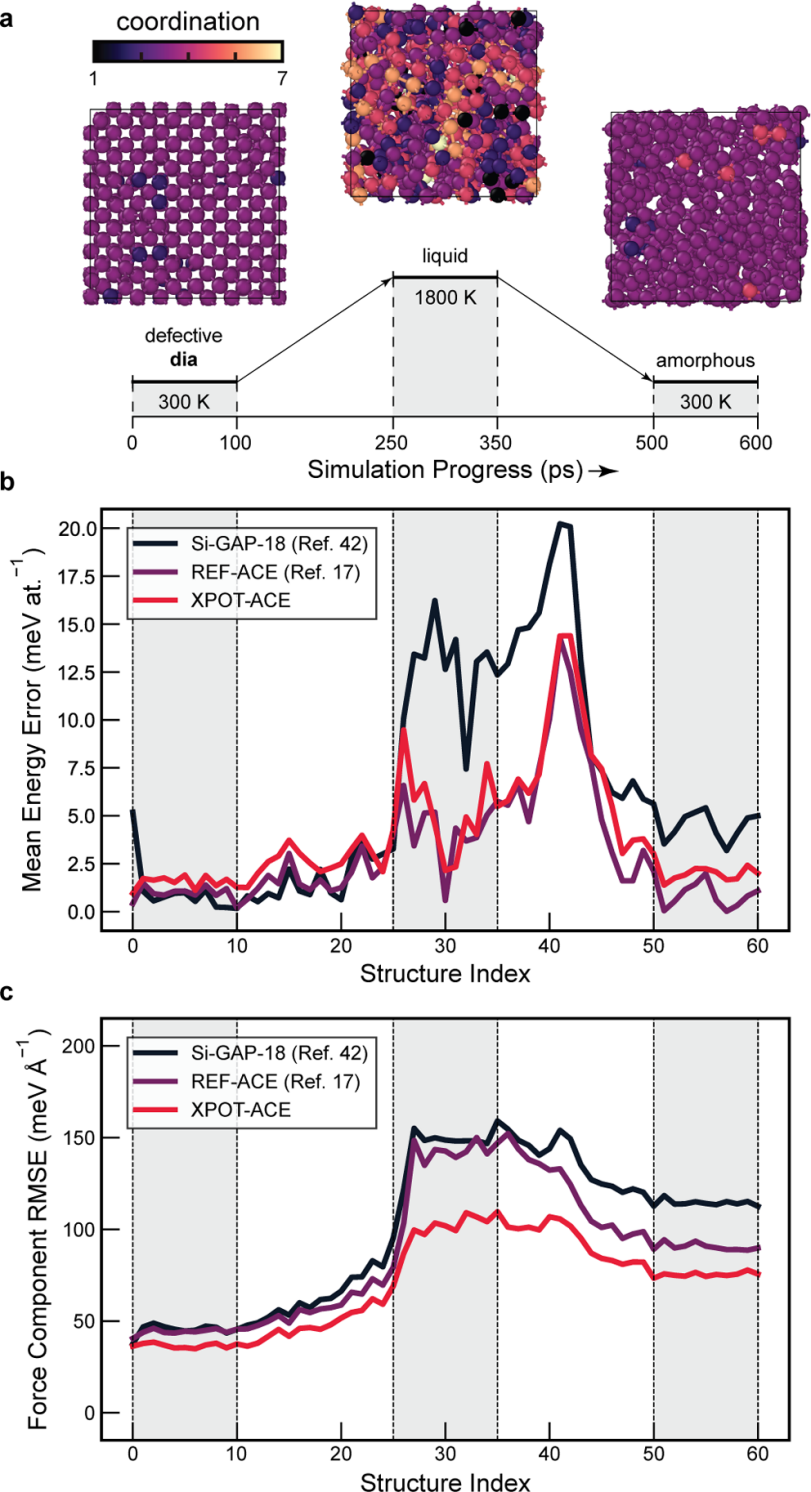
Accuracy of silicon ML potentials evaluated on DFT-labeled snapshots from a Si-GAP-18-driven melt–quench simulation reported in ref (42)., and visualized in the style of that prior work. (a) An overview of the constant-pressure simulation protocol, adapted from ref (42). The images show the three classes of structure seen at each stage of the simulation, color-coded according to coordination number. DFT snapshots were computed every 10 ps throughout the simulation. (b) Energy errors compared to DFT snapshots along the simulation trajectory. (c) Force errors for the same structures. XPOT-ACE outperforms all other potentials studied here in terms of force errors, but trails in energy errors to the ACE from ref (17). The schematic in panel (a) and the overall style are adapted from ref (42)., which is published under a CC BY license (https://creativecommons.org/licenses/by/4.0/).

We plot the errors for each structural snapshot from the MQ-MD test set in Figure 4. Doing so provides a more nuanced view of the types of configurations for which the potential shows higher or lower errors.^42^ We show that XPOT-ACE improves the force predictions compared to REF-ACE, while having very slightly higher errors (to within 1 meV at.^–1^). Our potential is comparatively most improved for the higher-energy liquid structures, while improvements for crystalline and amorphous structures are reduced, which we presume to be due to the uniform structure weighting. Notably, the higher weighting of the crystalline phases for existing potentials^17,35^ offers improved accuracy for crystalline configurations, whereas our potential still improves the force accuracy for those.

Across the simulation snapshots, the same structures are resulting in “spiking” (high error) energies across all three models. This suggests some underlying characteristic of the Si-GAP-18 training data set results in these less accurate predictions across models, especially on freezing of the liquid state (which is not strongly represented in the training data). For the latter, all three potentials show increased energy errors. The fluctuations are much reduced in force predictions, but there is still a visible “bump” in these predictions for the same structures.

Next, we quenched 500-atom randomized structures at fixed rates from 10^14^ to 10^11^ K s^–1^. Both XPOT-ACE and REF-ACE potentials were used and we compared the energies of the structures produced. On top of labeling these structures with XPOT-ACE, REF-ACE, and Si-GAP-18, we compute DFT energies for the structures, to quantify the predictive accuracy of all three potentials (akin to our studies of quenched SiO_2_ in ref (49)). For each rate, 5 random structures were quenched, with both mean and standard deviation reported in Table 2. We tested faster quench rates, but found that rates of 10^14^ and 10^15^ K s^–1^ led to structures within 2 meV at.^–1^ of each other for all models. We therefore do not include results for quench rates of >10^14^ K s^–1^ in Table 2.

Although the energies of structures for 10^12^ and 10^13^ K s^–1^ are very similar, XPOT-ACE predicts structures with slightly lower energies for 10^11^ K s^–1^, suggesting a more relaxed and therefore more stable a-Si structure. All potentials provide a good match to DFT. When compared to our findings in Table 1 and Figure 4, this is in line with our expectations.

Finally, we performed structural validation tests for the compression of silicon, as described in ref (47). This test was comprised of compressing a 100,000 atom low density amorphous silicon model up to 20 GPa. The pressurization rate is 0.1 GPa ps^–1^, and the temperature is held at 500 K. We observed similar behavior to what was seen in simulations using Si-GAP-18,^47^ whereby low-density amorphous (LDA) silicon upon compression collapses into a very-high-density amorphous (VHDA) phase at ≈12 GPa, and subsequently simple-hexagonal (sh) crystallites nucleate and grow. This result demonstrates that our XPOT-ACE model has learned much of the same behavior as Si-GAP-18 from the training set.

**Figure 5:**
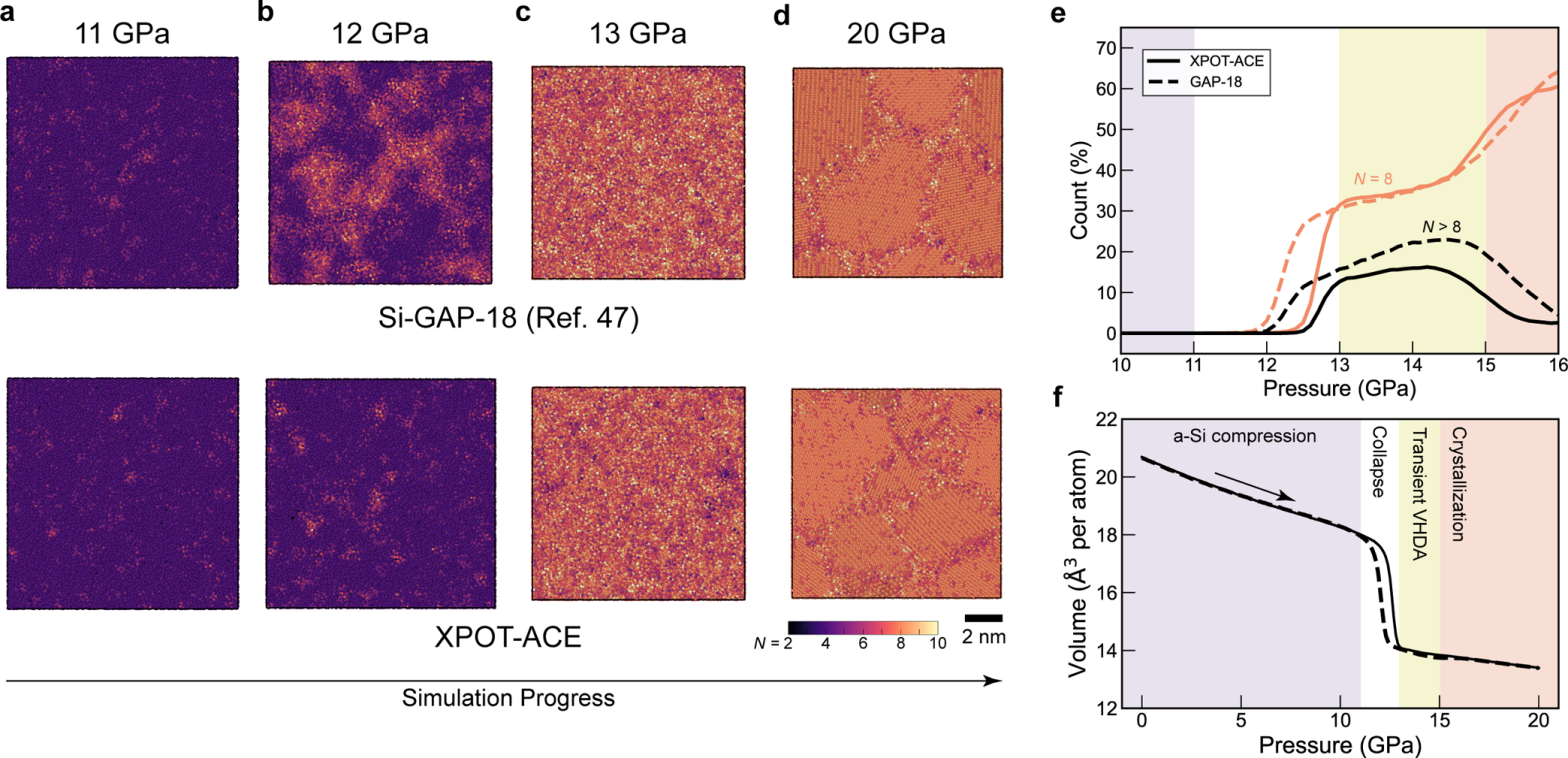
(a–d) Simulations of amorphous silicon under isothermal compression with both XPOT-ACE-2 (“XPOT-ACE” for brevity) and Si-GAP-18,^35^ similar to the simulations reported in ref (47). from which data for the GAP simulation are taken. Both potentials predict a collapse into VHDA occurring between 12–13 GPa from which simple hexagonal crystallites then form. (e) Coordination numbers as a function of pressure in the trajectories (determined by counting neighbors up to 2.85 Å). The initial increase in *N* > 8 atoms corresponds to the formation of the VHDA phase before crystallization occurs. (f) Volume against pressure during the simulations. Simulations were carried out using LAMMPS.^48^ The results are visualized in a similar way as in ref (47).

The formation of the VHDA phase in the compression simulation the using XPOT-ACE potential occurred at a pressure 0.5 GPa higher than that observed for Si-GAP-18, as shown by the relative lack of highly coordinated silicon atoms in Figure 5b—an effect which is corroborated by Figure 5e, where the percentage of *N* = 8 atoms does not rise substantially until 12.5 GPa, in contrast to the Si-GAP-18 simulation. Aside from this slightly delayed formation of VHDA, the potentials predicted almost identical densities throughout the simulation, and show behavior that is consistent with experiments for both VHDA formation^50^ and crystallization.^51^

### Antimony Telluride

To test our approach for a more complex material system, we fitted a potential for Sb_2_Te_3_, which is an important chalcogenide material used in various phase-change materials (PCM)-based devices for ultrafast data storage^54^ and high-performance neuromorphic computing tasks.^55,56^ PCMs have long served as key application cases for ML potentials, including early work on the binary material GeTe^57^ and the ternary Ge_2_Sb_2_Te_5_.^58^ The former potential has been used for studies of crystallization^59^ and thermal properties of GeTe;^60^ the latter has been applied to study structure and bonding in Ge_2_Sb_2_Te_5_^61^ and tested for the binary Sb_2_Te_3_.^62^

Our previous work has introduced an ML potential based on the GAP framework for Ge–Sb–Te (GST) alloys located along the compositional tie-line between GeTe and Sb_2_Te_3_.^7^ This GAP model, which we call “GST-GAP-22″, can accurately describe disordered structures of GST alloys and complex phase transition processes under practical programming conditions (e.g., nonisothermal heating) on the length scale of real-world devices.^7^ We took a subset of the GST-GAP-22 data set, which only contains elemental crystal structures of Sb and Te as well as binary bulk structures (including crystalline, amorphous, and intermediate crystallization configurations) found in the Sb–Te system.

A validation set was created using the protocol described in the Methods section, providing a representative sample of structures, and allowing us to consistently quantify the performance and robustness of our potentials. This is important for defining the loss function in XPOT (cf. eq 5), and so the validation set is fixed for all potentials fitted during optimization.

Additionally, to confirm that the reduction in scope of the training set (from the full Ge–Sb–Te system to only the Sb–Te system) was not unfairly advantaging our own optimized potentials, we fitted a GAP with the same hyperparameters as for the GST-GAP-22 potential,^7^ but using only the Sb–Te subset of the data for training. We use this potential (“SbTe-GAP” in the following) as a benchmark as it provides very similar force errors to GST-GAP-22, but resulted in improved accuracy for energy prediction.

**Figure 6:**
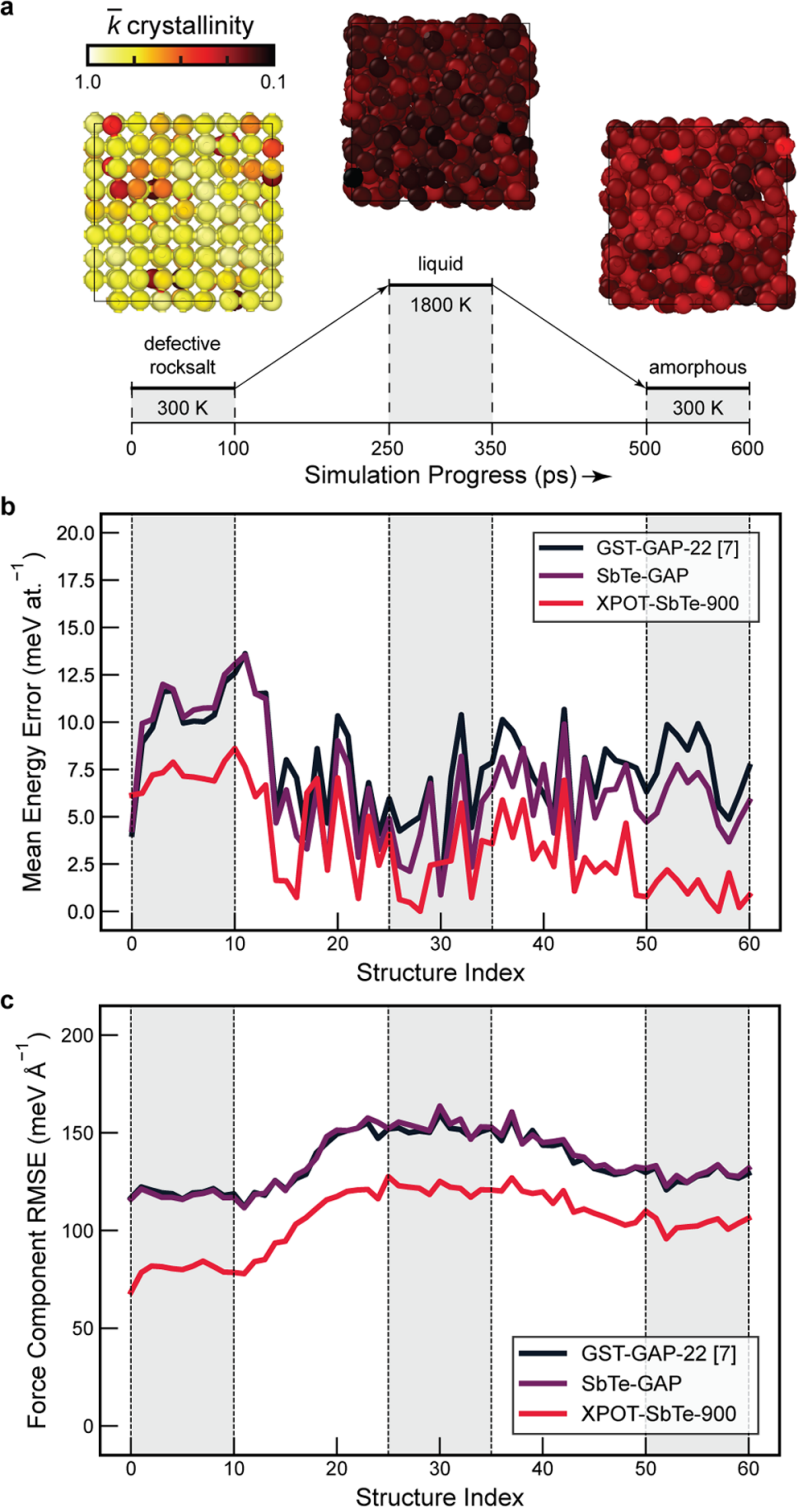
Prediction accuracy of Sb_2_Te_3_ ML potentials across DFT-labeled snapshots from a GST-GAP-22 melt-quench simulation. (a) An overview of the MD-based benchmark protocol created in a way similar to ref (42)., now for Sb_2_Te_3_. Structures show the three classes of structures seen at each stage of the simulation, color-coded according to a per-atom crystallinity measure.^52,53^ Snapshots were labeled with DFT every 10 ps throughout the simulation. (b) Energy errors compared to DFT snapshots across the simulation trajectory. (c) Force errors across the same structures. XPOT-ACE outperforms both GAP potentials on both energy and force errors.

In this case, we cannot directly compare to GST-GAP-22 numerically, as the validation data are taken from the GST-GAP-22 training data set. Therefore, to quantify the accuracy of the potentials, we created a new benchmark data set similar to that built for silicon in ref (42). (cf. Figure 4). Specifically, we performed a GAP-MD simulation in which Sb_2_Te_3_ was melted starting from a defective rocksalt-like crystal with an anion (Te) vacancy, before being quenched at 10^13^ K s^–1^ to form the amorphous phase. This trajectory was then labeled with DFT to produce a benchmark set representing crystalline, liquid, and amorphous Sb_2_Te_3_. Snapshots of these three phases are shown in Figure 6a. The atoms are color-coded by crystallinity as defined by the Smooth Overlap of Atomic Positions (SOAP)-based similarity,^52^ with respect to rocksalt-like Sb_2_Te_3_, as discussed in ref (53).

In Figure 6, we show that our XPOT-optimized ACE potential has improved not only upon the GST-GAP-22 predictions, but also the SbTe-GAP fitted on the Sb–Te subset of the full GST-GAP-22 database. The numerical accuracy is improved, and predictions are over 400× faster than for the GAP potentials. Figure 6b shows that the energy errors on the defective crystalline structures are consistently higher than for the liquid or amorphous phases across all potentials, with the same structural snapshots showing higher errors in this region. This occurs due to the relatively small number of crystalline structures with vacancy defects in the training database, and while our potentials all predict the energy to within 15 meV at.^–1^, we see that these structures present more of a challenge to these ML potentials than the liquid or amorphous structures do. The SbTe-XPOT-ACE potential is significantly more accurate than both GAPs in predicting energies and forces for amorphous structures, and the force predictions are uniformly over 10% closer to DFT than those of by either GAP potential.

**Figure 7:**
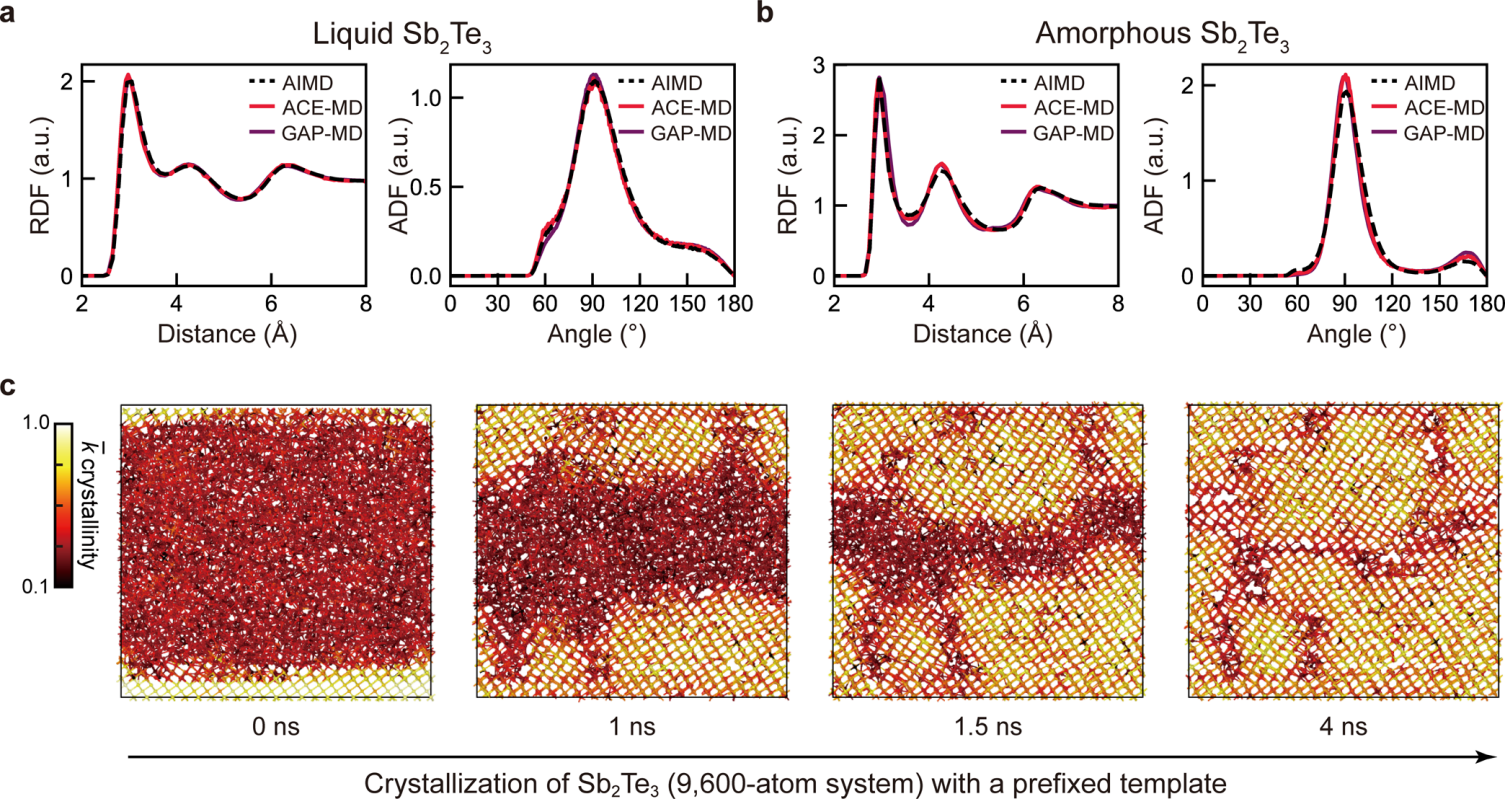
Structure and dynamics of Sb_2_Te_3_. (a–b) RDF and ADF plots for liquid and amorphous structures simulated by the SbTe-XPOT-ACE (red) and GST-GAP-22 (purple) models as well as AIMD (black). The data for the latter two are taken from ref (7). (c) Snapshots of an MD crystal-growth simulation. SOAP similarity is used to highlight the growth of crystalline Sb_2_Te_3_ (yellow) across snapshots. Several crystal grains and grain boundaries are visible. Simulations were carried out using LAMMPS.^48^

We carried out further physically guided validation, testing the similarity of structural predictions between AIMD, GST-GAP-22, and our XPOT-ACE potential, aiming to verify that numerical errors correspond to well-described physical properties and processes in simulation. We first characterize the structure of amorphous Sb_2_Te_3_. We ran a melt-quench simulation on a crystalline Sb_2_Te_3_ model containing 360 atoms. In Figure 7a–b, we show that our liquid and amorphous structures generated using SbTe-XPOT-ACE closely match the RDF and ADF of structures generated using AIMD and GST-GAP-22, including the shoulder in the ADF of the liquid phase. Both GST-GAP-22 and the XPOT-ACE potential marginally overorder the amorphous phase of Sb_2_Te_3_, evidenced by a slightly larger peak at 90° in the ADF and a larger second peak of the RDF, as compared to the AIMD reference (Figure 7b).

To move beyond structural validation, we produced a 9600-atom structural model of amorphous Sb_2_Te_3_ (in a box of 4.3 × 9.0 × 8.5 nm^3^) with a prefixed crystalline template to simulate the crystal-growth process. Figure 7c shows a crystallization simulation for this templated structural model. As in Figure 6, we used SOAP-based similarity^52^ with respect to rocksalt-like Sb_2_Te_3_ to quantify the per-atom crystallinity during the crystallization process.^53^ Our structural model was annealed at 600 K for 4 ns, and the growth proceeded quickly at the rough crystalline–amorphous interface. Upon nanosecond crystallization, we found many defects (e.g., point defects and layer stacking faults) in the recrystallized model (cf. the dark red atoms in Figure 5c), indicating competing growth of different crystalline regions with different crystal orientations. We note that such local disorder is challenging to fully characterize due to the short time scales on which they happen (e.g., in the programming operations of real-world devices), and high-temperature annealing can help to eliminate the local defects, e.g., via vacancy ordering,^63^ resulting in an energetically more favorable crystalline phase with fewer defects.^64^

## Conclusions

Hyperparameter optimization can improve the accuracy and efficiency of ML potentials. Alongside data set construction and fitting architectures, the choice of hyperparameters remains an important aspect of fitting performant ML models. Well-chosen hyperparameters can lead to more accurate, and more efficient, ML potentials for accelerated materials modeling.

In the present work, we have described an extension of our openly available XPOT code to ACE model fitting via PACEMaker.^17,18^ We have shown example applications for Si and Sb_2_Te_3_, two systems with diverse chemistry, across a wide range of configurational space (including liquid, amorphous, and crystalline phases). We thoroughly validated these potentials using a number of numerical and physical tests, and we believe that those types of benchmarks can be useful for other systems as well (see also ref (41)). In particular, expanding on ref (30)., we studied how suitable different types of test sets are to assess the quality of ML potentials across a range of structures—including “external” test sets that are different from the validation set used during optimization.

As ML potentials become larger and more complex, selecting suitable hyperparameters for fitting has become increasingly important to maximize performance and accuracy for a given training data set. Furthermore, our results in Table 1 suggest that series of potential fits with systematically varied hyperparameters could help to diagnose possible areas of failures for candidate ML potentials: specifically, the XPOT-ACE-3 model has low numerical errors on static MQ-MD snapshots but fails when used to drive MD itself, and this may be correlated with an extremely high prediction error on the RSS test set, which is generated distinctly from both the physically motivated MQ-MD and test data sets. In this way, series of XPOT runs might help users to judge whether more data are required, or whether the model hyperparameters require improvement. We hope for this work to contribute to the wider uptake of ML potentials and their application to increasingly challenging research questions in chemistry and materials science.

## Data Availability

The XPOT Python code is openly available via GitHub at https://github.com/dft-dutoit/xpot. XPOT includes interfaces to third-party software, including PACEmaker(17) which is freely available for academic noncommercial use. Data and code to reproduce the results from the present paper will be provided openly upon journal publication.

## Supporting Information Available

Additional numerical results and technical information as a supplement to Table 1, and hyperparameter ranges used for optimization (PDF)
