# Supplementary material for: Hyperparameter Optimization for Atomic Cluster Expansion Potentials
Source: J Chem Theory Comput. 2024 Nov 6;20(22):10103–13. doi: 10.1021/acs.jctc.4c01012 (PMC11603601; doi:10.1021/acs.jctc.4c01012)
Supplement: Supplementary file 1 — ct4c01012_si_001.pdf [file ct4c01012_si_001.pdf]

Supporting Information: Hyperparameter Optimization for Atomic Cluster Expansion Potentials

Daniel F. Thomas du Toit, Yuxing Zhou, and Volker L. Deringer*

Inorganic Chemistry Laboratory, Department of Chemistry, University of Oxford, Oxford OX1 3QR, UK

E-mail: volker.deringer@chem.ox.ac.uk

Energy and Force Errors for Silicon

In Table S1, we show a comparison of the energy and force errors for each dataset. Upon optimization, we see an improvement in both energy and force prediction accuracy for the testing dataset across all values of P, excluding the XPOT-ACE-3 potential as documented in the main text.

In improving the energy accuracy evaluated on the testing set (through optimization), we observe increases in the energy error for the MQ-MD dataset, and inconsistent prediction accuracy on the RSS dataset. The prediction errors on the RSS dataset show that optimizing on the Si-GAP-18 test set does not necessarily improve the accuracy across all structures, and that in some cases the energy accuracy of predictions for the RSS dataset worsens despite simultaneously improving in accuracy on the MQ-MD dataset. However, for $P \leq 2$, the RSS accuracy is always improved upon further optimization. The results for the "4F" potentials further emphasize that there is not a direct link between force and energy error improvements.

Table S1: Energy and force RMSE values of silicon potentials either upfitted from the best initial potential (within the first 4 iterations, labelled Initial) or upfitted from the final optimised hyperparameters (labelled XPOT-ACE). The errors are evaluated on three different test sets. The number for the models refers to the number of atomic properties, P, from linear (1) to quaternary (4), excluding XPOT-ACE-6827, labeled after the number of functions in the potential. As in the main text, the test sets used are the Si-GAP-18 test set of Ref. S1 ("GAP-18" for brevity), the melt–quench (MQ) MD test set of Ref. S2, and the random-structure-search (RSS) test set of Ref. S3, respectively.

			Energy RMSE (meV at.$^{-1}$)			Force RMSE (meV Å^{-1})		
	P	# Func.	GAP-18[S1]	MQ-MD[S2]	RSS[S3]	GAP-18	MQ-MD	RSS
XPOT-optimized models with increasing complexity								
Initial-1	1	3000	4.2	4.0	28.7	76	110	160
XPOT-ACE-1	1	3000	3.5	5.1	27.1	69	105	158
Initial-2	2	3000	6.5	3.5	192	70	104	1171
XPOT-ACE-2	2	3000	2.5	5.0	23.1	63	97	150
Initial-3	3	3000	4.8	3.3	42.8	65	97	296
XPOT-ACE-3	3	3000	318	5.2	$> 10^6$	300	98	$> 10^8$
Initial-4	4	3000	31.9	3.6	55.5	66	97	345
XPOT-ACE-4	4	3000	4.8	5.5	62.6	63	99	274
XPOT-optimized models with varied numbers of functions								
Initial-3F	3	1375	8.0	3.8	50.9	71	104	224
XPOT-ACE-3F	3	2000	4.6	4.1	20.5	65	97	139
Initial-4F	4	875	3.7	5.4	47.0	71	103	240
XPOT-ACE-4F	4	1625	2.5	5.4	72.5	64	100	187
Reference values								
XPOT-ACE-6827	1	6827	3.0	4.4	34.5	63	104	179
REF-ACE (Ref. S4)	1	6827	3.2	4.3	42.1	77	124	175
Si-GAP-18 (Ref. S1)	—	—	1.6	8.5	34.9	83	139	177

Optimized Hyperparameters

In Table S2, we provide the hyperparameter ranges over which we carried out optimization for the current work on silicon. The ranges here were determined by a small number of pilot studies (for the hyperparameters `functions_per_element`, `rcut`, and `dcut`). For the remaining hyperparameters, we studied existing literature values and defaults, and took ranges that represented a window around these values, as we aim to test the ability for XPOT optimisation to provide improvements in performance for users without significant experience with a particular fitting method.

Table S2: Hyperparameters optimized during the XPOT iteration process for Si. These values are also available in the input files in the associated GitHub repository.

Hyperparameter	Optimization Range
`rcut`	5–8
`dcut`	0.001–0.1
`radbase`	'SBessel', 'ChebExpCos', 'ChebPow'
`radparameters`	1–10
`fs_parameters`	0.1–10
Where number of functions optimized ("-F" models):	
`functions_per_element`	500–2000

In Table S3, we provide the same details for the Sb_2Te_3 potentials. These values take the learnings from Si, and uses them to refine the optimization ranges and values. This results in a reduced number of parameters (where we found optimal values), and an increased range in certain others. We also do not optimize number of functions, as during pilot tests, 900 functions per element provided the accuracy-to-performance ratio we desired for this work.

Table S3: Hyperparameters optimized during the XPOT iteration process for Sb_2Te_3. These values are also available in the input files in the associated GitHub repository.

Hyperparameter	Optimization Range
`rcut`	3.8–8
`dcut`	0.001–1
`radparameters`	1–10
`fs_parameters` (exponents)	0.025–0.975

MD Speed Testing

MD speed testing was performed in LAMMPS[S5] using the ML-QUIP (for GAP[S6]) and ML-PACE[S4] implementations that were the latest available as of January 2024 and compiled together. A standard simulation of 4,096 atoms of a-Si at 300 K was used to validate the computational cost of each potential. No outputs were required during the simulation, via files or standard output, in order to minimize computational overhead. Each simulation ran on a single core of an AMD EPYC 7763 processor using a single MPI thread. The ACE potentials used the recursive algorithm, as implemented by default in the `pace` pair style, and the GAP model was run using the `quip` pair style.

References

(S1) Bartók, A. P.; Kermode, J.; Bernstein, N.; Csányi, G. Machine Learning a General-Purpose Interatomic Potential for Silicon. *Phys. Rev. X* **2018**, *8*, 041048.

(S2) George, J.; Hautier, G.; Bartók, A. P.; Csányi, G.; Deringer, V. L. Combining Phonon Accuracy with High Transferability in Gaussian Approximation Potential Models. *J. Chem. Phys.* **2020**, *153*, 044104.

(S3) Morrow, J. D.; Deringer, V. L. Indirect learning and physically guided validation of interatomic potential models. *J. Chem. Phys.* **2022**, *157*, 104105.

(S4) Lysogorskiy, Y.; van der Oord, C.; Bochkarev, A.; Menon, S.; Rinaldi, M.; Hammerschmidt, T.; Mrovec, M.; Thompson, A.; Csányi, G.; Ortner, C.; Drautz, R. Performant implementation of the atomic cluster expansion (PACE) and application to copper and silicon. *npj Comput. Mater.* **2021**, *7*, 97.

(S5) Thompson, A. P.; Aktulga, H. M.; Berger, R.; Bolintineanu, D. S.; Brown, W. M.; Crozier, P. S.; in 't Veld, P. J.; Kohlmeyer, A.; Moore, S. G.; Nguyen, T. D.; Shan, R.; Stevens, M. J.; Tranchida, J.; Trott, C.; Plimpton, S. J. LAMMPS - a flexible simulation tool

for particle-based materials modeling at the atomic, meso, and continuum scales. *Comput. Phys. Commun.* **2022**, *271*, 108171.

(S6) Bartók, A. P.; Payne, M. C.; Kondor, R.; Csányi, G. Gaussian Approximation Potentials: The Accuracy of Quantum Mechanics, without the Electrons. *Phys. Rev. Lett.* **2010**, *104*, 136403.
